# The association between depression and alcohol use among pregnant adults in the USA

**DOI:** 10.1007/s00737-023-01417-x

**Published:** 2024-01-10

**Authors:** Madison Chapman, Gretchen Bandoli, Shira M. Goldenberg

**Affiliations:** 1 Department of Pediatrics, University of California San Diego, La Jolla, CA USA; 2 Division of Epidemiology and Biostatistics, School of Public Health, San Diego State University, San Diego, USA

**Keywords:** Prenatal alcohol use, Fetal alcohol spectrum disorders, Depression, Mental health

## Abstract

**Background:**

The prevalence of alcohol use among pregnant women aged 18–44 years old increased in recent years. The influence of mental health issues on an individual’s likelihood to use alcohol during pregnancy has not been thoroughly investigated. This study will examine the association between experiencing a major depressive episode (MDE) in the past year and past-month alcohol use among pregnant women using the 2011–2020 National Survey on Drug Use and Health (NSDUH).

**Methods:**

Pregnant women between the ages of 18 and 44 years old were included in the study for analysis. Multivariable logistic regression analysis was used to examine the association between past-year MDE and past-month alcohol use adjusting for age, race/ethnicity, marital status, and employment status. Additional logistic regression analysis was performed to investigate whether this relationship differed by trimester of pregnancy.

**Results:**

A total of 6745 participants were included in the analytic sample. The prevalence of past-year MDE and past-month alcohol use was 7.67% and 9.15% respectively. Logistic regression analysis showed past-year MDE was significantly associated with past-month alcohol use in pregnant women adjusting for age, race/ethnicity, marital status, and employment status (aOR = 1.96; 95% CI, 1.34–2.87). This relationship became stronger in second and third trimesters of pregnancy.

**Conclusions:**

This study showed a positive association between MDE and past-month alcohol use among pregnant women, with strongest effect estimates in the third trimester. These findings may inform approaches for improved screening guidelines and health education for individuals who may be at higher risk of prenatal alcohol use.

## Introduction

Prenatal alcohol exposure can cause significant physical, behavioral, and learning disabilities commonly referred to as fetal alcohol spectrum disorders (FASDs). Types of FASDs include fetal alcohol syndrome, alcohol-related neurodevelopmental disorder, alcohol-related birth defects, and neurobehavioral disorder associated with prenatal alcohol exposure (CDC 2022a). A person with an FASD may experience symptoms such as abnormal facial features; low body weight; poor coordination; hyperactivity and attention deficits; speech and language delays; heart, kidney, and/or bone defects; shorter-than-average height; and small head size. In addition to the myriad of health effects caused by FASDs, there is also significant economic burden on the individual with an FASD and the US healthcare system. It is estimated that fetal alcohol syndrome alone costs the USA over $4 billion annually. Maternal characteristics that are associated with prenatal alcohol use include unmarried marital status, ethnicity, tobacco use, older age, employment status, illicit drug use, and parity (Denny 2019; Meschke et al. 2003; Rubio et al. 2008). Between 2011 and 2018, the prevalence of alcohol use among pregnant women aged 18–44 years old increased from 9.2 to 11.3% (Denny et al. 2020). Ample research has shown associations between prenatal alcohol use and FASDs, highlighting the need for further research that will educate individuals who may become pregnant about the risks associated with prenatal alcohol use and improve screening practices during prenatal appointments to prevent or minimize alcohol use in pregnancy.

To date, there have been very few studies that examine the effect of mental health disorders like depression on prenatal alcohol use. Studies that have investigated the association of depression and prenatal alcohol use have either not used widely accepted diagnostic criteria for a major depressive episode (MDE), such as that of the Diagnostic and Statistical Manual of Mental Disorders (DSM) or did not assess MDE that that was experienced within the past year (Leis et al. 2012; Meschke et al. 2003; Flynn and Stephen 2008).

It is estimated that over 10% of adults in the USA have experienced major depressive disorder (MDD) in the past year, and over 20% have experienced MDD at some point in their lifetime (Hasin et al. 2018). The prevalence of lifetime MDD is nearly twice that in women than men (21.3% versus 12.7% respectively), and this disparity is theorized to be associated with several factors including hormonal mechanisms, major life events and transitions related to pregnancy and childbirth, and the disproportionate burden of social-structural marginalization borne by women including gender-based violence and disadvantaged social status (Noble, 2005). Qualitative data from a 2018 study suggest women feel favorable towards receiving mental health screenings during obstetric appointments, but reported issues with securing mental health treatment and services following screenings (Byatt et al. 2018). Additional research has shown that while 8.2% of pregnant women screened positive for depression, only 12% of these women reported receiving mental health treatment within the past year (Byatt et al. 2016). These findings highlight the need for consistent mental health screenings during prenatal appointments as well as better access to adequate follow-up care.

By identifying maternal risk factors for prenatal alcohol use, we can target modifiable factors to ultimately prevent FASD while promoting an integrative approach to addressing mental health care and alcohol misuse to benefit both mother and child. Therefore, the objective of this study was to evaluate the association between past-year major depressive episode and past-month alcohol use among pregnant women in the USA aged 18–44 years old, using the 2011–2020 National Survey on Drug Use and Health.

## Methods

### Study design and setting

The National Survey on Drug Use and Health (NSDUH) was started in 1971 and has been conducted annually in the USA since 1990 for the purpose of measuring the prevalence of substance use and mental health issues. The sample for this survey was selected using an independent, multistage area probability sample design (Department of Health and Human Services 2021). The sample was made up of civilian, noninstitutionalized individuals from all 50 states and the District of Columbia aged 12 years and older at the time of the survey. The surveys were conducted using computer-assisted interviews and audio computer–assisted self-interviews. In 2020, in-person interviews were limited, and web-based screening and interview procedures were implemented due to the COVID-19 pandemic. The current study utilized cross-sectional data from the NSDUH public use datafiles for the years 2011 through 2020.

### Study population

**Fig. 1:**
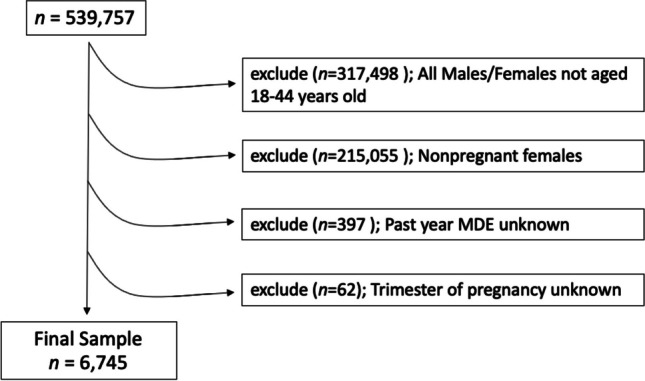
Study population inclusion criteria flowchart

The population for the current study included pregnant US women between the ages of 18 and 44. A total of 539,757 individuals were included in the combined 2011–2020 NSDUH public use data files. All women who were under the age of 18 or over the age of 44, men, and nonpregnant women were excluded from the study sample (*n* = 532,553). Additionally, those with missing responses for the primary exposure, past-year major depressive episode (*n* = 397), and those missing responses for trimester of pregnancy (*n* = 62) were excluded, resulting in a final analytic sample of 6745 participants (Fig. 1).

### Data variables/ measurement tools

#### Outcome

The primary outcome for the current study was defined as any alcohol use within the past 30 days. The survey question to assess this measure asked, “How long has it been since you last drank alcohol?” Initial response options included “within the past 30 days,” “more than 30 days ago but within the past 12 months,” “more than 12 months ago,” “used at someone in the past 12 months,” and “used at some point in the lifetime.” Responses were recoded and dichotomized to include those who had consumed alcohol within the past 30 days, and those who had not.

#### Exposure

Major depressive episode (MDE) was assessed based on criteria outlined by the DSM 4–5. Respondents were defined as having MDE if they responded affirmatively to at least five of nine depression-related symptoms in the same 2-week period in which at least one of the symptoms was a depressed mood or loss of interest or pleasure in daily activities. These nine symptoms included depressed most of the day, markedly diminished interest or pleasure in all or almost all activities most of the day, changes in weight, insomnia or hypersomnia, psychomotor agitation or retardation, fatigue or loss of energy, feelings of worthlessness, diminished ability to think or concentrate or indecisiveness, and recurrent thoughts of death or recurrent suicide ideation. Individuals were classified as having MDE in the past year if they met these criteria within the past year.

#### Covariates

Potential confounders were chosen a priori upon a review of previous literature. Age (Tan et al. 2015), race/ethnicity (Denny et al. 2020), marital status (Tan et al. 2015), and employment status (Denny et al. 2020) were considered for analyses based on the findings of prior studies that reported associations between the covariates, MDE, and alcohol use.

### Statistical methods

For all analyses, sampling weights (provided by NSDUH), stratification, and clustering were applied to ensure nationally representative findings. Descriptive statistics including frequencies and crude prevalence estimates were calculated for all variables of interest. Bivariate analyses were performed to determine crude unadjusted associations between selected characteristics and past-month alcohol use. Multivariable logistic regression was used to investigate the relationship between MDE and past-month alcohol use while adjusting for selected covariates. All covariates identified a priori were included in the final model. An additional stratified multivariable analysis was conducted to investigate whether the association between MDE and past-month alcohol use varied by trimester of pregnancy. All analyses were performed using SAS software version 9.4 (SAS Inc., Cary NC).

## Results

### Demographics

The weighted and unweighted frequencies and percent distributions of study participants by demographic characteristics are displayed in Table 1. Among the study population, 617 (9.15%) reported alcohol use in the past 30 days, and 517 (7.67%) experienced MDE within the past year.

**Table 1:** Characteristics of pregnant women aged 18–44 from the 2011–2020 National Survey on Drug Use and Health

Characteristic	Unweighted		Weighted		
	*n*	%	*n*	%	(SE)
	6745	100	1,069,190	100	
Past-year major depressive episode					
No	6228	92.34	997,490	92.33	(0.4)
Yes	517	7.66	71,700	7.67	(0.4)
Past-month alcohol use					
No	6128	90.83	971,137	90.85	(0.5)
Yes	617	9.17	98,054	9.15	(0.5)
Age					
18–20	976	14.47	95,227	8.91	(0.5)
21–25	2769	41.05	258,775	24.20	(0.6)
26–34	2418	35.85	558,408	52.22	(0.9)
35–44	582	8.63	156,781	14.66	(0.8)
Race/ethnicity					
White	3716	56.38	602,787	56.38	(0.9)
Black or African American	1059	15.48	165,528	15.48	(0.8)
Spanish/Hispanic/Latina	1300	18.79	200,909	18.79	(0.7)
Other	670	9.34	99,967	9.35	(0.6)
Marital status					
Married	3514	52.10	650,050	60.80	(0.9)
Divorced, separated, or widowed	358	5.31	59,004	5.52	(0.4)
Never married	2873	42.59	360,136	33.68	(0.9)
Education level completed					
≤ High school	3026	44.86	388,167	36.30	(0.8)
Some college/Associate Degree	2011	29.81	304,033	28.44	(0.8)
≥ Undergraduate degree	1708	25.32	376,991	35.26	(1.0)
Employment status					
Employed full-time	2650	39.29	475,992	44.52	(0.9)
Employed part-time	1224	18.15	180,925	16.92	(0.7)
Not employed part or full-time	2871	42.56	412,274	38.56	(0.9)

The majority of participants were 26–34 years old (52.22%) and non-Hispanic White (56.38%), followed by Hispanic (18.79%) or Black (15.48%). Participants were more likely to be married (60.80%) and employed full-time (44.52%). Among participants, 36.30% had a high school degree or less, 28.44% had completed some college or an associate degree, and 35.26% had attained an undergraduate degree or higher. The prevalence of past-year MDE in the sample was 7.67%, and the prevalence of past-month alcohol use was 9.15%.

### Bivariate analysis

In a bivariate analysis, the odds of past 30-day alcohol use were higher among those who experienced past-year MDE (odds ratio 1.90, 95% CI 1.31-2.74), were aged 35–44 years old (OR 1.36, 95% CI 0.95–1.96), Black or African American race/ethnicity (OR 1.27, 95% CI 0.93–1.74), and divorced, separated, or widowed (OR 2.14, 95% CI 1.34–3.43) compared to those who did not experience past-year MDE (Table 2).

**Table 2:** Bivariate logistic regression analysis by past-month alcohol use among pregnant women aged 18–44 years old

Characteristic	Past-month alcohol use (weighted *n* = 99,990)		No past-month alcohol use (weighted *n* = 979,900)		Bivariate associations
	Weighted *n*	% (SE)	Weighted *n*	% (SE)	OR (95% CI)
Past-year major depressive episode					
Yes	11,311	11.31 (1.8)	61,803	6.31 (0.3)	1.90 (1.31–2.74)
No	88,679	88.69 (1.8)	918,097	93.69 (0.2)	1.00 (Reference)
Age					
18–20	7297	7.30 (1.3)	89,317	9.11 (0.5)	0.85 (0.56–1.30)
21–25	22,871	22.87 (1.9)	238,516	24.34 (0.7)	1.00 (Reference)
26–34	51,356	51.36 (2.7)	510,723	52.12 (1.0)	1.05 (0.82–1.34)
35–44	18,467	18.47 (2.4)	141,345	14.42 (0.8)	1.36 (0.95–1.96)
Race/ethnicity					
White	60,209	60.21 (2.5)	545,492	55.67 (1.0)	1.00 (Reference)
Black or African American	20,546	20.55 (2.3)	146,180	14.92 (0.8)	1.27 (0.93–1.74)
Spanish/Hispanic/Latino	13,364	13.37 (1.8)	191,855	19.13 (0.8)	0.63 (0.47–0.85)
Other	5872	5.87 (1.2)	96,374	9.8 (0.7)	0.55 (0.34–0.90)
Marital status					
Married	53,061	53.07 (2.8)	601,186	61.35 (1.0)	1.00 (Reference)
Divorced, separated, or widowed	9,497	9.50 (1.9)	50,190	5.12 (0.4)	2.14 (1.34–3.43)
Never married	37,433	37.44 (2.6)	328,524	33.53 (0.9)	1.29 (1.02–1.63)
Education level completed					
≤ High school	32,582	32.59 (2.5)	361,515	36.89 (0.9)	1.00 (Reference)
Some college/Associate Degree	29,052	29.06 (2.5)	277,758	28.35 (0.9)	1.16 (1.16–1.17)
≥ Undergraduate degree	38,356	38.36 (3.7)	340,627	34.76 (1.1)	1.25 (0.95–1.64)
Employment Status					
Employed full-time	56,005	56.01 (2.4)	424,645	43.34 (0.9)	1.00 (Reference)
Employed part-time	15,919	15.92 (1.8)	165,841	16.92 (0.8)	0.73 (0.53–0.99)
Not employed part or full-time	28,066	28.07 (2.3)	389,414	39.74 (0.9)	0.55 (0.43–0.69)

### Multivariable analysis

In a multivariable analysis adjusted for a priori selected confounders (age, race/ethnicity, marital status, and employment status), compared to those who did not experience past-year MDE, the adjusted odds of past-month alcohol use were significantly higher among those who experienced past-year MDE (AOR 1.96, 95% CI 1.34–2.87) (Table 3).

**Table 3:** Multivariable logistic regression model for past-month alcohol use among pregnant women aged 18–44 years old, NSDUH, 2011–2020 (*n* = 6745)

	Multivariable Association		
	Adjusted odds ratio	95% confidence interval	*P*
Past-year major depressive episode			
No	1.00	Reference	
Yes	1.96	1.34–2.87	0.0006

Model is adjusted for age, race/ethnicity, marital status, and employment status

**Fig. 2:**
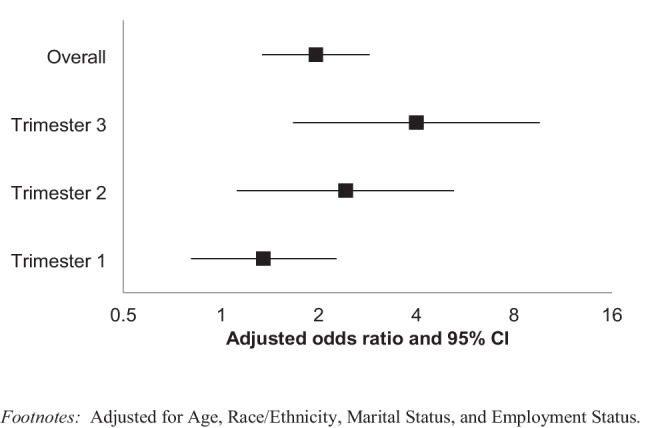
Association between past-year major depressive episode and past-month alcohol use by trimester among pregnant women aged 18–44 years old, NSDUH, 2011–2020, (*n* = 6,745). Adjusted for age, race/ethnicity, marital status, and employment status

In a secondary analysis stratified by trimester of pregnancy, the association between MDE and alcohol use was significantly influenced by trimester (Fig. 2; Table 4). There was no association between MDE and prenatal alcohol use in trimester 1 (aOR 1.35, 95% CI 0.81–2.27), but association was observed in trimester 2 (aOR 2.42, 95% CI 1.12–5.22) and trimester 3 (aOR 4.00, 95% CI 1.67–9.68). As displayed in Fig. 2 and Table 4, the association becomes stronger as trimester increases, indicating a possible dose response relationship.

**Table 4:** Association between past-year major depressive episode and past-month alcohol use among pregnant women aged 18–44 years old by trimester, NSDUH, 2011–2020 (*n* = 6745)

Trimester	Past-year major depressive episode	Past-month alcohol use (weighted *n* = 99,990)		No past-month alcohol use (weighted *n* = 979,900)		Adjusted OR (95% CI)
		Weighted *n*	%	Weighted *n*	%	
1	Yes	6669	9.80	20,265	7.61	1.35 (0.81–2.27)
	No	61,343	90.20	246,197	92.39	
2	Yes	2548	13.61	23,688	6.34	2.42 (1.12–5.22)
	No	16,174	86.39	350,149	93.66	
3	Yes	1986	17.54	16,545	5.00	4.00 (1.67–9.68)
	No	9334	82.46	314,293	95.00	

## Discussion

### Principal findings

In this nationally representative study of US women from 2011 to 2020, 9.15% of pregnant women reported past-month alcohol use, and 7.66% were found to have experienced a major depressive episode within the previous year. Having experienced MDE within the past year was significantly associated with alcohol use among pregnant women after adjusting for age, race/ethnicity, marital status, and employment status (aOR = 1.96 95% CI 1.34–2.87). The relationship between MDE and alcohol use was significantly influenced by the trimester of pregnancy, with the relationship becoming significantly stronger in the second and third trimesters.

### Comparison to other studies

The prevalence of past-month alcohol use estimated in this study is similar to previous US data. For example, a 2017 study using data from the National Survey on Drug Use and Health, 2005–2014 found that 8.7% of pregnant women aged 18–44 years old reported any alcohol use in the past month (Oh et al. 2017). Although the 2017 study did not report the prevalence of past-year MDE among all pregnant women, they did report this prevalence among pregnant women who reported alcohol use in the past 30 days. They found that 9.2% of pregnant women who reported alcohol use in the past 30 days were found to have experienced MDE in the past year, compared to 11.3% found in this study. Although this study also showed a positive relationship between past-month alcohol use and past-year MDE among pregnant women (aOR 1.55, 95% CI 0.99–2.42), this finding was not statistically significant in their population. Differences in findings between the current study and the comparison study may be due to different study years, particularly as a result of increasing prevalence of MDE in recent years (Goodwin et al. 2022).

Other studies have shown varying results in regard to the association between depression and alcohol use in pregnancy. Our findings are consistent with several previous studies which have shown a positive association between depression and alcohol use among pregnant women (Meschke et al. 2003; Pajulo et al. 2001; de Jesus Silva et al. 2016). However, others found no significant association (Oh et al. 2017; Leis et al. 2012).

A prospective cohort study from the UK that assessed the relationship of anxiety and depression symptoms and prenatal alcohol use found anxiety to be significantly associated with alcohol use, but depression was not significantly associated with alcohol use (Leis et al. 2012). The population for this study consisted of mainly white women from a small town in the UK, and demographic differences may partially explain differences in the findings with the current study. Depression was measured in this study at 18 weeks’ gestation using a 10-item assessment called the Edinburgh Postnatal Depression Scale. Alcohol use was measured at 32 weeks’ gestation by asking respondents the amount of alcohol they regularly consumed at present time, in contrast to the current study which defined alcohol use as any amount of alcohol consumed at any point within the past month. These differences in the measurements of depression and alcohol use may explain some of the variation in the findings of the current study and the comparison study.

### Strengths and limitations

This study has several limitations. First, alcohol use was self-reported, which may have resulted in underestimation due to the social desirability bias related to reporting alcohol use during pregnancy. Additionally, this study used cross-sectional data and had limited covariates for multivariable adjustment; therefore, we are unable to determine a causal relationship between MDE and alcohol use. Also, in estimates limited to the first trimester, we cannot determine whether alcohol use overlapped with pregnancy awareness, although given the 30-day use and the average gestational age at pregnancy recognition at 5.5 weeks, we assume that most exposure likely did. Finally, we were unable to study drinking patterns associated with MDE, including binge drinking, which has been identified as a specific risk factor for FASD (Watson and Angelotta 2022; Roozen et al. 2018). Despite these limitations, this study has several strengths. Very few studies have characterized the association between MDE and prenatal alcohol use while adjusting for sociodemographic characteristics. This study contributes to the limited base of literature on the association between mental health disorders and alcohol use during pregnancy. This study leveraged data from NSDUH which provided a nationally representative sample, and results are generalizable to all civilian, non-institutionalized individuals within the USA, excluding only military personnel and incarcerated populations.

### Implications

The findings of this study indicate that MDE is associated with alcohol use during pregnancy. Although a temporal relationship cannot be confirmed due to the cross-sectional nature of this study, depression was measured by symptoms that occurred within the past year, while alcohol use was measured by use within the past month; therefore, it can be speculated that depression may have been present before the alcohol use occurred. An important finding of this study was that the relationship between depression and alcohol use became stronger in the second trimester and was strongest in the third trimester. A longer duration of prenatal alcohol exposure has been identified as conferring greater risk of adverse infant outcomes (Bandoli et al. 2019). Therefore, assessing this relationship stratified by trimester was important because it suggests factors associated with alcohol use in pregnancy may change depending on the time of its occurrence, which may help accurately target screening and direct interventions.

### Recommendations

While screening and counseling for prenatal alcohol use, special consideration should be given to patients who have a history of depression or suspected depression. The CDC estimates that 20% of women are not screened for depression during prenatal appointments, although the American College of Obstetrics and Gynecology (ACOG) recommends all patients be screened at least once for perinatal depression (CDC 2022b; ACOG 2017). If depression status is unknown, greater emphasis should be placed on depression screening during these appointments because of the increased prevalence of alcohol use among patients with depression. Additionally, it is imperative to not only emphasize depression screenings during prenatal appointments, but also to ensure pregnant women have access to adequate and affordable mental health services.

Future research focused on establishing a causal relationship between depression and prenatal alcohol use is needed, including prospective studies as well as future studies addressing other mental health disorders such as anxiety. Additionally, social contexts should be carefully considered in future research as life events such as parity, loss events, domestic violence, social isolation, and other forms of trauma are intimately connected with both mental health status and ones propensity to consume alcohol. The use of biomarkers could also aid in the accurate measurement of alcohol use, such as 5-hydroxytrptophol (5-HTOL), ethyl glucuronide (EtG), and fatty acid ethyl esters (FAEE), which are direct byproducts of ethanol and may be reliable options for use in future studies (Ghosh et al. 2019).

## Conclusion

In summary, this study found a significant positive association between MDE and past-month alcohol use among pregnant women aged 18–44 years old, for which the strength increased in later pregnancy. These findings may inform approaches for improved screening guidelines and health education for individuals who may be at higher risk of prenatal alcohol use. Further longitudinal research is needed to comprehensively examine the relationship between mental health issues and prenatal alcohol use.

## Data Availability

All data are publicly available at https://www.samhsa.gov/data/data-we-collect/nsduh-national-survey-drug-useand-health.

## Appendix

**Table:**

Codebook		
Variable description	Response options	Final categorization
Outcome		
Past-month alcohol use		
“How long has it been since you last drank alcohol?”	Within the past 30 days More than 30 days ago but within the past 12 months More than 12 months ago Used at some point in the past 12 months Used at some point in the lifetime	Yes—used within the past 30 days No—did not use within the past 30 days
Key exposure		
Past-year major depressive episode		
“Did you feel sad, empty, or depressed most of the day nearly every day?” “Did you feel discouraged about how things were going in your life most of the day nearly every day?” “Did you lose interest in almost all things like work and hobbies and things you like to do for fun?” “Did you lose the ability to take pleasure in having good things happen to you, like winning something or being praised or complimented?” “Did you have a much smaller appetite than usual nearly every day during that time?” “Did you have a much larger appetite than usual nearly every day?” “Did you gain weight without trying to during that [worst/most recent] period of time?” “How many pounds did you gain?” “Did you lose weight without trying to?” “How many pounds did you lose?” “Did you have a lot more trouble than usual falling asleep, staying asleep, or waking too early nearly every night during that [worst/most recent] period of time?” “During that [worst/most recent] period of time, did you sleep a lot more than usual nearly every night?” “Did you talk or move more slowly than is normal for you nearly every day?” “Were you so restless or jittery nearly every day that you paced up and down or couldn’t sit still?” “During that [worst/most recent] period of time, did you feel tired or low in energy nearly every day, even when you had not been working very hard?” “Did you feel that you were not as good as other people nearly every day?” “Did you feel totally worthless nearly every day?” “During that [worst/most recent] period of time, did your thoughts come much more slowly than usual or seem confused nearly every day?” “Did you have a lot more trouble concentrating than usual nearly every day?” “Were you unable to make decisions about things you ordinarily have no trouble deciding about?” “Did you often think about death, either your own, someone else’s, or death in general?” “During that period, did you ever think it would be better if you were dead?” “Did you think about committing suicide?” “In the past 12 months, did you have a period of time when you felt [FEELNOUN] for two weeks or longer while also having some of the other problems we asked about?”	Yes No	Yes – Past-year major depressive episode No – No-past year major depressive episode
Covariates		
Age		
Date of birth	Date of Birth	18–20 years old 21–25 years old 26–34 years old 35–44 years old
Race/ethnicity		
Recoded—race/Hispanicity	Non-Hispanic White Non-Hispanic Black/African American Non-Hispanic Native American/Alaskan Native Non-Hispanic Native Hawaiian Non-Hispanic Asian Non-Hispanic more than one race Hispanic	Non-Hispanic White Non-Hispanic Black/African American Hispanic Other (includes non-Hispanic Native American/Alaskan Native, non-Hispanic Native Hawaiian, non-Hispanic Asian, non-Hispanic more than one race)
Marital status		
Imputation revised marital status	Married Widowed Divorced or separated Never been married	Married Divorced, separated, or widowed Never married
Educational attainment		
Education—recoded imputation revised	Less than high school High school graduate Some college/Associate degree College graduate	Less than or equal to high school diploma Some college/Associate degree Greater than or equal to undergraduate degree
Employment status		
Employment status—imputation revised	Employed full time Employed part time Unemployed Other (including not in labor force)	Employed full time Employed part time Not employed part or full time (includes unemployed and other (including not in labor force))
